# Spontaneous triple vessel cervicocephalic artery dissection in a young gentleman: A case report

**DOI:** 10.5339/qmj.2024.6

**Published:** 2024-02-15

**Authors:** Liaquat Ali, Khawaja Haroon, Naveed Akhtar, Khalid Zammar, Ahmad Meer, Majd Abualrob, Isra Eltazi, Zeba Noorain, Yahya Baniamer, Randa Yasin

**Affiliations:** Department of Neurology, Hamad General Hospital, Hamad Medical Corporation, Doha Qatar; Department of Neurology, Weill Cornell Medicine, Doha, Qatar Email: lali5@hamad.qa

**Keywords:** Multiple simultaneous cervicocephalic dissections, dissecting aneurysm (pseudoaneurysm), fibromuscular dysplasia (FMD), intracranial hemorrhage (ICH)

## Abstract

Introduction: Cervicocephalic arterial dissections (CADs) occur in 3 cases per 100,000 individuals across all ages. Multiple simultaneous CADs are found in 13 to 22% of cases, and three or more dissections occur in approximately 2%. CADs might result from multifactorial intrinsic deficiencies of vessel wall integrity and extrinsic factors, e.g., minor trauma.

Case Presentation: A young gentleman presented to the emergency department with a sudden onset of a spinning sensation of surrounding, left side arm weakness, blurring of vision, and an NIHSS score of 4. An urgent CT scan of the head and intracranial angiogram showed bilateral severe stenosis of the distal cervical segment of internal carotid arteries (ICAs) and right vertebral artery moderate stenosis at the V2 segment. He had been given IV TPA (Alteplase) within the 4.5-hour window. After 4 hours, the patient’s GCS dropped from 15 to 10, and the NIHSS score increased from 4 to 24, followed by witnessed a generalized tonic-clonic seizure. Repeat urgent CT head showed no evidence of intracerebral hemorrhage (ICH). The patient was arranged for cerebral angiographic catheterization that showed bilateral flame-shaped occlusion of cervical ICA dissection. There is a mild focal narrowing of the right cervical vertebral artery, likely dissection. Routine laboratory blood workup for vasculitis was negative. During MICU admission, he had witnessed the right arm hemichorea-ballism spectrum abnormal movement. After the 6th-month follow-up, intracranial CT angiogram showed reduced caliber of the bilateral distal cervical course of the internal carotid arteries seen with residual dissection and focal outpouching of the right ICA representing pseudoaneurysm.

Discussion: The occurrence of multiple CADs suggests the presence of an underlying intrinsic arteriopathy, such as FMD, the presence of pseudoaneurysm, environmental triggers, cervical manipulation, and remote history of head or neck surgery. A study of the most extensive case series of patients with cervical artery dissection showed 15.2% of patients with multiple CAD. In most patients with multiple cervical artery dissections, antithrombotic treatment is effective, complete recanalization, and the outcome is favorable. Outside the window period of acute ischemic stroke, either anticoagulation or antiplatelet therapy is a recognized treatment for secondary ischemic stroke prevention due to extracranial artery dissection. For acute stroke or TIA patients caused by intracranial artery dissection, experts recommend antiplatelet therapy rather than anticoagulation.

Conclusion: Simultaneous triple-vessel cervicocephalic arterial dissections are rarely reported condition. Multiple CADs are associated with underlying vasculopathy and environmental triggers, and a majority are recanalized with antithrombotic treatment with favorable outcomes. Antithrombotic treatment is effective in most patients with multiple CADs, and most expect complete recanalization. This case report guides physicians in the treatment and outcome of acute stroke due to multiple CAD.

## Introduction

This paper presents Qatar’s first rare case report of spontaneous triple-vessel cervicocephalic artery dissections (CADs). Stroke is the second most common cause of morbidity and mortality globally. Acute ischemic stroke accounts for 62% of all incident strokes worldwide. The lifetime risk of stroke for adult men and women (>25 years) is approximately 25%.^1^ CADs are a common cause of stroke in young patients but may occur at any age. In studies from North America and Europe, the mean age of individuals affected by dissection was 48.5 years (range 44 to 53), and the prevalence of dissection as a cause of ischemic stroke is higher in younger adults.^2-4^ Dissection of the cervical and cerebral arteries occurs in about 3 cases per 100,000 individuals across all ages yet accounts for up to one-quarter of all strokes in the young.^2^ When arterial wall structural integrity is compromised, it results in CAD, allowing blood to collect between layers as an intramural hematoma. Spontaneous dissection is labeled as a result without overt trauma, although there is often a triggering event or arteriopathy. According to North American and European reports, extracranial dissection is far more frequent than intracranial dissection. However, evidence from several case series suggests that intracranial dissection is more common in Asian populations and children.^5,6^ Extracranial carotid dissections mainly occur 2 cm or more above carotid bifurcations, while intracranial carotid dissections are most commonly in the supra-clinoid segment. Vertebral artery dissection primarily occurs in the foraminal segment from cervical transverse processes of C6 to C2 (V2 segment) or the extracranial extradural segment between the transverse process of C2 and the foramen magnum at the base of the skull (V3 segment).^7,8^ Multiple simultaneous CADs are found in 13 to 22% of cases. Three or more CADs occur in approximately 2% of cases. In various reports, the rate of recurrent ischemic stroke and Transient Ischemic Attack (TIA) after dissection ranges from 0 to 13%.^9^ The prospective CADISS trial found that the rate of recurrent ischemic stroke at three months was approximately 2%, and all recurrences were within ten days of randomization, suggesting that the risk beyond the first two weeks is very low.^10^ When patients complain of intense unilateral posterior cervical and occipital pain or temporal pain, CADs should be suspected.^11^ The diagnosis of dissection is confirmed by neuroimaging findings, particularly the demonstration of a long tapered arterial stenosis or occlusion, a dissecting aneurysm (pseudoaneurysm), an intimal flap, a double lumen, or an intramural hematoma.^12-14^

## Case Presentation

**Figure 1.**
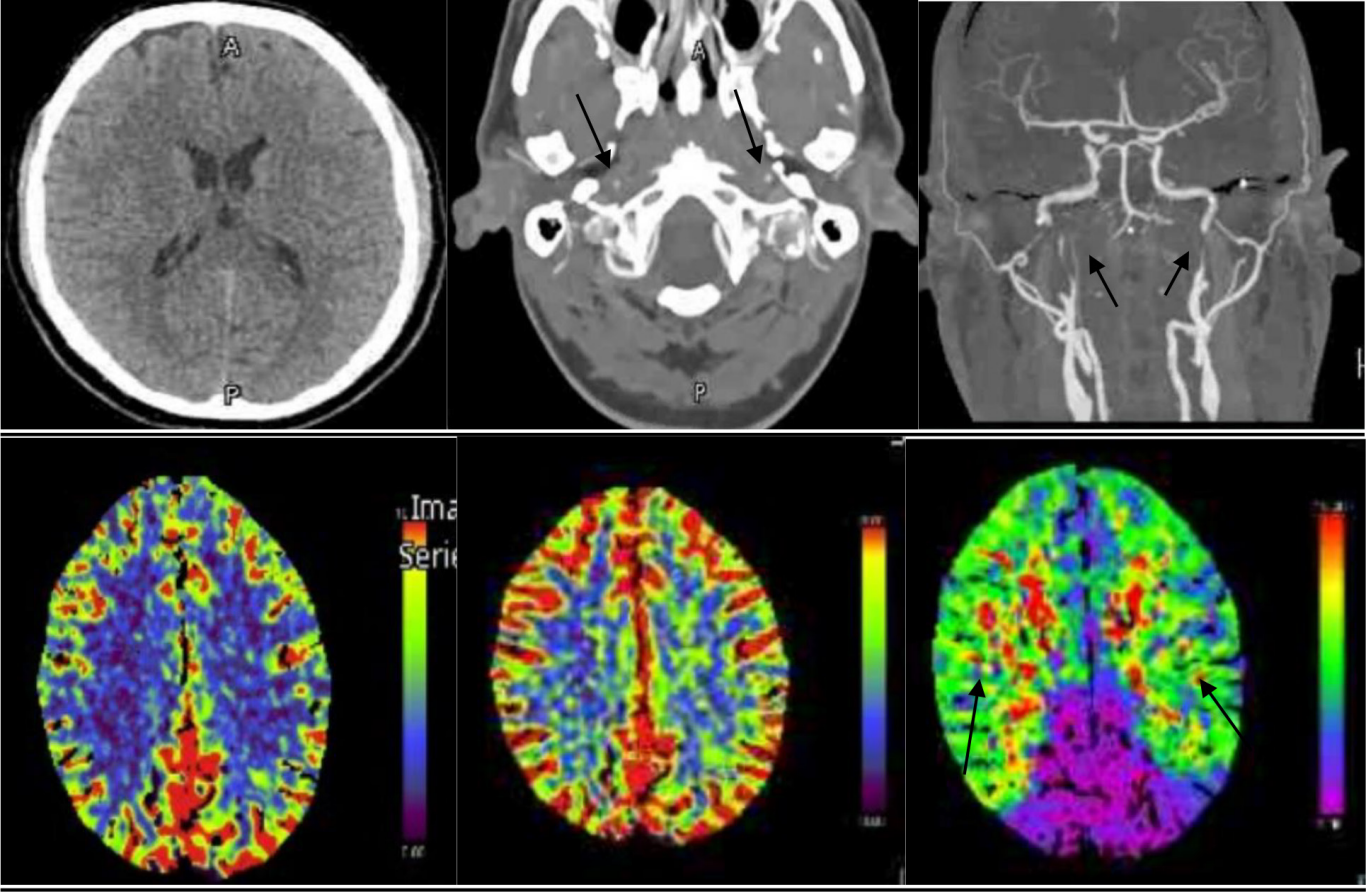
CT head showed normal brain parenchyma attenuation and no territorial hypodensity. CT perfusion showed symmetrical bilateral perfusion abnormality involving the anterior circulation with increased time to drain (TTD), likely secondary to severe stenosis of the distal cervical segments of the ICAs bilaterally. CT angiogram showed bilateral severe stenosis noted at the distal cervical segment of the bilateral ICAs and moderate stenosis at the V2 segment of the right vertebral artery.

**Figure 2.**
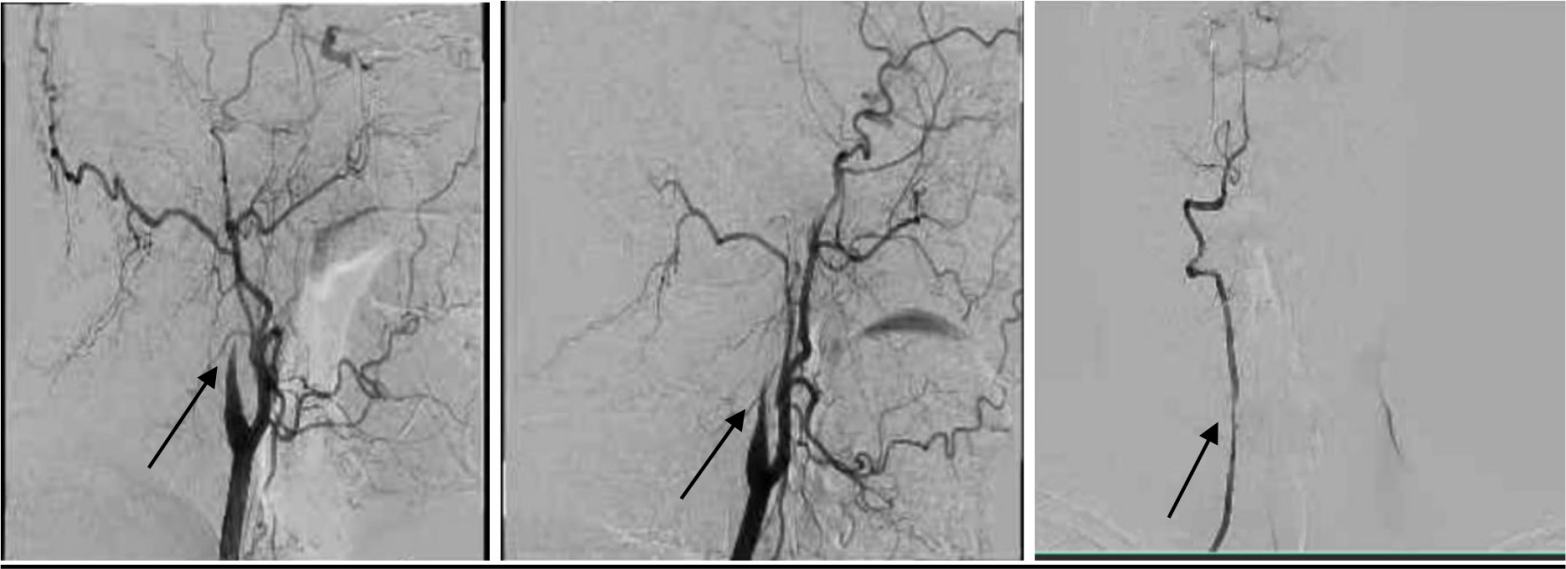
Cerebral angiogram selective catheterization showed bilateral flame-shaped signs of occlusion, implicating both cervical ICAs typical for dissection. Right ICA is revascularized via ECA/ophthalmic artery anastomosis. The left ICA has good revascularization through the left PCOM and the ophthalmic /ECA anastomosis. There is a mild focal narrowing of the cervical RT vertebral artery, likely dissection.

A 40-year-old man without any prior medical history of chronic comorbidities arrived at the emergency department (ED) experiencing a sudden onset of acute spinning sensation of his surroundings, left-sided arm weakness, blurring of vision, and an unsteady gait at 7 am. He woke up at around 6 am, called his friend for help, and arrived at the ED after 55 minutes with a National Institutes of Health Stroke Scale (NIHSS) score of 4. The patient’s friend mentioned that he was confused and had slurred speech. The patient has smoked one pack of cigarettes per day for 22 years and has alcohol consumption; both are risk factors for acute stroke and carotid dissection. An urgent Computed Tomography (CT) scan of the head, CT perfusion, and CT angiogram were performed that showed bilateral severe stenosis of the long segment (2 cm) of the distal cervical segment of Internal Carotid Arteries (ICAs), moderate right vertebral artery stenosis of a long segment (2.5 cm) at the V2 segment, and bilateral symmetrical increase time to drain (TTD) and maintained cerebral blood volume (CBV) in the perfusion study as shown in Figure 1. He had been given an intravenous Tissue Plasminogen Activator (Alteplase) within the window. At 11 am, the patient’s Glasgow Coma Scale (GCS) dropped from 15 to 10, and the NIHSS score increased from 4 to 24. The patient had witnessed a generalized tonic-clonic seizure, and he received lorazepam and levetiracetam. Urgent repeat CT head was requested to rule out intracranial hemorrhage (ICH), and there was no evidence of ICH. An Interventional Neuroradiologist was consulted, cerebral angiographic catheterization was performed and showed bilateral flame-shaped signs of occlusion implicating both cervical ICA typical for dissection. These long dissecting segments measure around 6-7 cm in length. There is a mild focal narrowing of the cervical right vertebral artery at the V2 segment, likely dissection, as shown in Figure 2.

**Figure 3.**
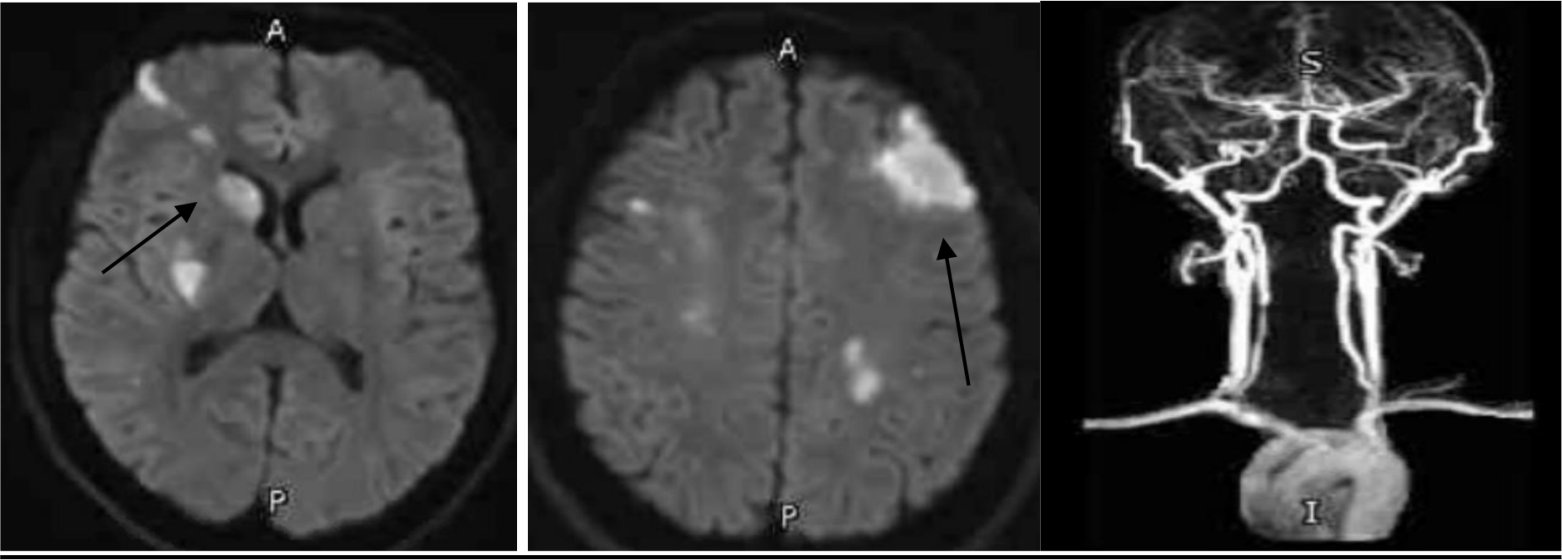
MRI, MRA head, and neck vessels showed multiple, variable-sized areas of acute ischemic strokes seen in the right frontal lobe, basal ganglia, corona radiata, and centrum semiovale along the MCA territory, and the similar regions are seen in the left frontoparietal lobes. MRA showed bilateral severe stenosis of the upper cervical ICA and cranial right ICA. The visualized parts of the right MCA and ACA are markedly attenuated. There is stenosis of the middle 1/3 of the cervical right vertebral artery.

The patient was admitted to the medical intensive care unit (MICU) for closed neuromonitoring. During the next 24 hours in MICU, the patient’s condition was stable without worsening neurological deficits. MRI brain, MRA brain, and neck vessels showed multiple variable-sized areas of acute ischemic strokes in the right frontal lobe, basal ganglia, corona radiata, and centrum semiovale along the MCA territory. Similar areas of acute ischemic stroke are seen in the left frontoparietal lobes—bilateral severe stenosis of the upper cervical and cranial segment of bilateral ICAs. The visualized parts of the right MCA and ACA are markedly attenuated. There was severe stenosis of the middle 1/3 segment of the right cervical (V2) vertebral artery, as depicted in Figure 3.

On the second day of MICU admission, repeated stereotyped abnormal movements of the right arm rising in the air and bringing to face were witnessed, most likely hemichorea-ballism spectrum, and a portable video EEG showed frequent random generalized slow background suggestive of diffused cerebral cortical dysfunction. Routine laboratory blood workup for vasculitis was negative, as detailed in Table 1. The patient was started on Aspirin, Atorvastatin, Levetiracetam, and Escitalopram. On the fourth day, the patient was stepped down to the stroke unit, followed by a transfer to the rehabilitation center.

**Figure 4.**
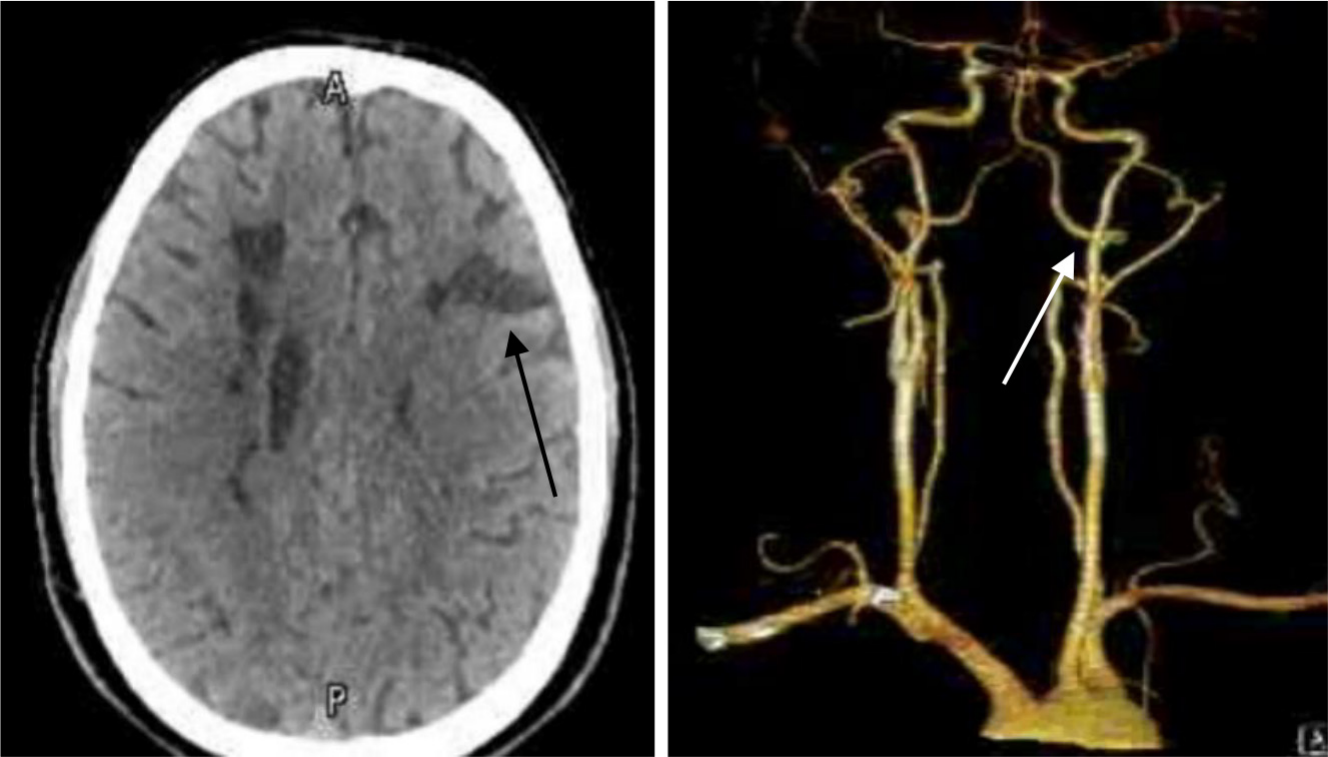
CT cerebral angiogram showed good filling of the bilateral common carotid arteries to the carotid bifurcation and the proximal cervical course of the internal carotid artery. The reduced caliber of the bilateral distal cervical course of the internal carotid arteries is seen with residual dissection, and focal outpouching of the right ICA is noted opposite C1 right lateral mass representing ICA pseudoaneurysm. The vertebral arteries bilaterally appear to be patent, with dominant left-sided noted.

After the 6th-month follow-up, intracranial CT angiogram showed focal outpouching of the right ICA opposite to the C1 level, representing ICA pseudoaneurysm as shown in Figure 4. The patient was discharged after five months from an adult daycare rehabilitation program with complete independence in eating, sphincter control, good comprehension, expression, and social cognition while modified independence in grooming, dressing, bathing, toilet, bed, and supervised climbing stairs with Functional Independence Measure (FIM) was 118 /126 (93.65%). Mini-mental State Examination (MMSE) score was 30/30.

## Discussion

CADs are a common cause of stroke in young individuals aged between 44 and 53, with multiple simultaneous CADs found in 13 to 22% of cases. Three or more CADs occur in approximately 2% of cases.^15,16^ Various reports indicate that the rate of recurrent ischemic stroke and TIA after dissection ranges from 0 to 13%.^17^ The occurrence of multiple spontaneous CADs suggests the presence of an underlying intrinsic arteriopathy, with a high frequency of pseudoaneurysms, possibly reflecting the fact that multiple dissections are more often sub-adventitial.^15^ However, in this case report, there is no evidence of fibromuscular dysplasia (FMD), hereditary connective tissue disease, potential precipitating events such as infection and/or minor neck trauma, or a family history of spontaneous CADs. Nonetheless, after five months, the follow-up intracranial CT angiogram showed focal outpouching of the right ICA, representing an ICA pseudoaneurysm.

A study by Bejot et al. presented the most extensive case series of patients with CAD. This study compared the baseline characteristics and short-term outcomes of patients with multiple to single CAD. Of 983 patients with CAD, 15.2% (149) presented with multiple CADs. Multiple CADs were more often associated with underlying vasculopathy such as fibromuscular dysplasia (OR, 3.97; 95% CI,2.04-7.74), presence of a pseudoaneurysm (OR,2.91;95% CI,1.88-4.57), and environmental triggers such as recent infection, cervical manipulation, and a remote history of head or neck surgery. However, in patients with multiple rather than single cervical artery dissection, there was no significant difference in function 3-month outcome with a modified Rankin scale (MRS) score of equal or greater than 3; 12% in multiple versus 11.9% in single (OR,1.20;95% CI,0.60-2.41).^18^

In another study by Hassan et al., a comparison of 76 patients’ clinical and angiographic features of single versus multiple spontaneous extracranial and/or intracranial arterial dissection was made. Multiple CADs were found in 10 patients (22%) who had a higher proportion of pseudoaneurysms, were more likely to involve the posterior circulation, had a higher prevalence of fibromuscular dysplasia, and were more prevalent in young individuals (<45 years) when compared to single arterial dissection.^15^ In a study by Janquli M et al. for the long-term outcome of 39 patients of CAD, clinical follow-up was available for 33 patients (84.6%), and the majority witnessed complete resolution of symptoms (30 patients, 90.9%).^19^ In most patients with multiple CADs, antithrombotic treatment is effective, complete recanalization is expected in 78% of dissected vessels, and a favorable long-term outcome and prognosis.^16^

For any patients with CAD presenting with acute ischemic stroke, standard approaches to stroke management should be evaluated to determine eligibility for reperfusion therapy with intravenous thrombolysis and/or mechanical thrombectomy. Outside the window period of acute ischemic stroke, either anticoagulation or antiplatelet therapy is a recognized treatment for secondary ischemic stroke prevention due to extracranial artery dissection. The limited available evidence suggests that there is no difference in efficacy between anticoagulation and antiplatelet therapy for ischemic stroke prevention in patients with extracranial dissection. However, anticoagulation is likely associated with a higher risk of hemorrhagic events.^20,21^ For acute stroke or TIA patients caused by intracranial artery dissection, experts recommend antiplatelet therapy rather than anticoagulation. In a few cases, dissection of intracranial arteries, which lack an external elastic lamina and have only a thin adventitial layer, may lead to vessel rupture with subarachnoid hemorrhage and a high risk of early rebleeding (as high as 40%) and early repair is typically recommended.^22^ Management is individualized according to location and other anatomic features and can include proximal occlusion of the artery, wrapping of pseudoaneurysm, bypass, embolization, or stenting. However, complicated endovascular interventions may cause additional morbidity.^23^

## Conclusion

This is a rare case report of spontaneous triple vessel CADs locally with a favorable outcome. Multiple CADs are associated with underlying vasculopathy and environmental triggers, and the majority are recanalized with antithrombotic treatment. This case report guides physicians in reperfusion therapy for acute stroke due to multiple CAD to enhance clinical relevance and a standard approach, and future large-scale multicentral studies would contribute to a more comprehensive understanding of the epidemiology, risk factors, management, and outcomes of CADs.

## Conflict of Interest

There is no conflict of interest.

## Authors’ Contributions

Liaquat Ali; data collection & analysis, manuscript writing & review literature. Khawja Hassan Haroon; data analysis, manuscript writing, manuscript review. Naveed Akhtar; data analysis, manuscript writing, manuscript review. Khalid Zammar; data analysis, manuscript writing, manuscript review. Ahmad Meer; manuscript writing, manuscript review Majd Abualrob; manuscript writing, manuscript review Isra Eltazi; manuscript writing & review literature Zeba Noorain; data collection Yahya Baniamer; review literature anda Yasin; manuscript review Data Availability: The authors declare that data supporting the findings of this study are available within the article.

## Ethical Approval

Protocol IRC ID MRC -04-23-741: entitled: Spontaneous triple vessel cervicocephalic artery dissections in a young gentleman, a case report, has been approved by MRC, HMC, Qatar. 

## Figures and Tables

**Table 1.** Levels of certain indicators in the patient compared to normal ranges.

**Indicators**	**(Normal ranges)**	**Patients result**
CRP	(0-5 mg/L)	3.4
ESR	(2-28 mm/hr)	20
ANA		Negative
ANCA		Negative
RA factor	(0-14 IU/ml)	< 10 IU/ml
C3 Complement level	(0.9-1.8 gm/L)	1.3 gm/L
C4 Complement level	(0.1-0.4 gm/L)	0.32 gm/L
Homocysteine level	(0-13 umol/L)	5.5umol/L
Fibrinogen level	(2-4.1gm/L)	3.94
D-dimer	(0-0.49 mg/L FEU)	< 0.30
HbA1c	(Desirable < 5.7%)	5.5%
TSH	(0.3-4.2 mlU/L)	3.25
Vit B12	(145-596 pmol/L)	362
Cholesterol	(< 5.2 mmol/l)	5.7
LDL	(< 2.59 mmol/l)	4.0
Triglyceride	(< 1.7 mmol/l)	1.7
Ethanol level	(0-10.8 mmol/L)	< 2.2 mmol/L
Treponema pallidum antibodies		Non-reactive
PT	(9.4-12.5 second)	11.8 second
APPT	(25-36 second)	29 second
Urea	(2.5-7.8 mmol/L)	3.8
Creatinine	(62-106 umol/L)	61
Troponin-T HS	(3-15 ng/L)	5 ng/L
